# Moral Elites and the De-Paradoxification of Danish Social Policy Between Civil Society and State (1849–2022)

**DOI:** 10.1007/s11266-022-00509-z

**Published:** 2022-08-10

**Authors:** Anders Sevelsted

**Affiliations:** 1 Department of Management, Politics, and Philosophy, Copenhagen Business School, Porcelænshaven 18, 4.132, 2000 Frederiksberg, Denmark; 2 School of Social Work, Lund University, Lund, Sweden

**Keywords:** Voluntarism, Welfare state, Philanthropy, Deservingness, Moral elites

## Abstract

The article argues that in Denmark during the past 150 years, moral elites have been central in settling paradoxes within social policy by developing ‘classifications’ of citizens and sectors: who are deserving of help and what sector (public or third) should provide care. Contrary to widely held beliefs, historically, there is no logical or practical connection between ‘more deserving’ and ‘state support’. Theoretically, the article integrates elite scholarship and cultural sociology in developing a concept of moral elites’ *power from*—their sources of moral authority—and *power to*, the way that they have used their power to classify citizens and sectors. Empirically, the Danish moral elite and its involvement in social policy are analyzed based on secondary as well as primary historical sources. Findings: The development of the Danish moral elite has roots in the administrators of the nineteenth-century absolutist state: the clergy, medical doctors, and lawyers. Educational resources and state affiliation continue to be central to moral elite status. Economists have ascended to the top of the moral elite, while clergymen have dropped out. Three major classifications were developed during the period. ‘Help to self-help’ (late nineteenth century): deserving poor should receive help from private charity, while the public system should deter and discipline. ‘Rights’ (mid-twentieth century): the state should care for all, philanthropy mostly considered stigmatizing. ‘Workfare’ (late twentieth century to present): citizens are considered deserving as long as they are ‘active’, and sectors are considered equal in providing for citizens as long as they reach the economistic goal of activation.

## Introduction

Western social policy (in the broadest sense of this term) is founded on a paradox, where philanthropic benevolence based in gift-giving and state-administered relief based on social rights from the nineteenth century have existed side by side as competitors and collaborators as paradoxical “contradictory yet interrelated elements that exist simultaneously and persist over time” (Smith & Lewis, 2011, p. 382). The paradox ‘trickles down’ so that recipients of public benefits or philanthropy are put in a situation where they are often confronted with multiple and conflicting demands (Åkerstrøm Andersen, 2008) or ‘scene styles’ (Lichterman & Eliasoph, 2014).

There are, however, groups that seek to create paradox-free social policies where criteria for deservingness and responsibilities for provision are clear: the moral elites. Unlike power elites who have access to resources such as money or direct political power, moral elites have access to normative power resources: Ideas, knowledge, or moral status derived from education, position in certain organizations like the church or voluntary organizations, or even personal charisma. What ‘counts’ as a resource for moral status varies over time. In nineteenth-century Denmark, the traditional moral elites of the absolutist state consisted mainly of members of the clergy, medical doctors, lawyers, and eventually economists. Many were elevated to moral elite status by virtue of their position in the state administration, but others gained recognition from being part of civil society organizations, reform commissions, or as the intellectuals of political classes. In present-day Denmark, moral elite status is still built on educational credentials and affiliation with the state and (to a lesser extent) influential NGOs, even as think tanks and a widened public sphere have emerged. In the article, I show how in the past 150 years, economists have taken over a central position among the moral elites at the expense of other groups.

Moral elites de-paradoxify social policy through acts of classification. Classification means deciding what groups are deserving of a benefit and what groups are not, as well as what sector should be responsible for caring for the groups in question. E.g., are the unemployed deserving of help (since they are not wholly responsible for their own situation)? And should they primarily be helped by the public system or civil society organizations?

In this article, I take a historical view of the case of Danish social policy history in order to show how changing moral elites have managed to de-paradoxify social policy. The article first reviews existing research to show how the paradox of social policy has been addressed so far. It then introduces the Danish case. The theoretical part of the article introduces the concept of moral elites as groups that have the authority to articulate moral principles of society. In the area of social policy, this authority has been exercised by developing social policy classifications that have classified target groups, relations, and institutions as belonging to civil or noncivil spheres. The analytical part of the article examines the de-paradoxifying social policy classifications of moral elites in three eras: a ‘Help to self-help classification’ (1849–1891), a Rights classification (1891–1976), and a Workfare classification (1976-present) in which moral elites struggled over classifications. The conclusion sums up various classification strategies deployed by changing moral elites and discusses theoretical and empirical implications.

## Deservingness and Sectors in the Literature

Much social policy literature seems to take for granted that questions of deservingness are closely related to questions of sector provision: The more target groups are considered deserving, the more state involvement is called for. However, historically the relationship between deservingness and sector provider has varied. In the nineteenth century, state support was thought to (and designed to) carry more stigma than private support, and consequently, it was reserved for the undeserving poor. Conversely, the deserving poor were supposed to get by on private relief. This relation is mostly forgotten in the literature.

Third sector civil society scholars tend to assign different roles to the two sectors. The third sector may act as a ‘first responder’, but does not have the capacity, professionalism, or impartiality of the state (Salamon, 1987). Conversely, welfare state scholars tend to focus on the welfare state as an almost teleological process of rights development (Marshall, 1992), changing power dynamics between social groups (Baldwin, 1999; Esping-Andersen, 1990; Korpi, 1983), or the realization of increased societal interdependence by elites (de Swaan, 1988). Somewhat relatedly, Marxians have described the emergence and disappearance of welfare policies as the result of contradictions and paradoxes in the economy (Alber, 1988; Gough, 1979; Harvey, 2007, 2015).

While all of these perspectives have value and point to different pieces of the puzzle of the emergence and roles of the modern welfare states, they all tend to downplay the ideational or ideological element of the relationship between public and private relief: Perceptions of deservingness *as well as perceptions of sectors* vary historically in much more complex ways than the literature acknowledges.

There are of course scholars who recognize the historically varying perceptions of deservingness. Deservingness theorists have developed a comprehensive approach to show how institutional arrangements and perceptions of certain target groups interact to support or undercut the social legitimacy of these groups (Oorschot et al., 2017). This literature also recognizes the stigma of poverty and of receiving social benefits (Gilens, 1999; Katz, 1990). The blind spot of these studies, however, is the third sector. The esteem of this sector as provider of benefits does not enter into the analysis of perceptions of deservingness.

However, what I term the social policy paradox cannot be understood comprehensively, if one does not consider the relationship between perceptions of sectors and perceptions of the deservingness of target groups. Historical analyses of the ideational foundation of social policy in Britain point out that not only groups, but also sectors can fall in and out of elites’ grace as views of the state and civil society on interpersonal relations change (Harris, 1992; Offer, 2003).

I argue that moral elites have been crucial in determining the relationship between sectors and deservingness status—and thus in handling the social policy paradox. This is reminiscent of de Swaan’s seminal work on the role of elites in overcoming the collective action problem as the result of taking into account their increased interdependence with the lower classes: poor laws, sewage systems, and social insurance come about as the result of this realization (de Swaan, 1988). Nonetheless, de Swaan focuses on elite groups’ objective material interests and does not show how certain elite groups were crucial in framing and legitimizing nationally varying types of social policy—even if there is clear evidence that public opinion is to a large extent shaped by elite discourse (Schneider & Jacoby, 2005). Deservingness studies are similarly mostly concerned with public support for social policy—and less interested in the groups that frame and shape policies.

## The Danish Case and Methods

The welfare classifications I explore in this article can be found in any developed, non-totalitarian welfare system: settlements between competing and contradictory logics that exist over time in the realm of social policy. I view the case of the development of the welfare classifications in Denmark as a single case study with inherent comparative qualities (Flyvbjerg, 2006)—even though the present study is not comparative. Denmark is a case of the development of social policy in the Nordic countries characterized by strong central states, high administrative capacity, cultural homogeneity, strong nineteenth-century social movements, as well as high free church/revivalist engagement in the social issues of the nineteenth century. The aim of the study is not explanatory, but analytically to show the de-paradoxifying efforts of changing moral elites. The Danish case is well described, enabling me to rely on a large material that describes how changing elites have settled the relationship between public and private relief historically. I rely on this already published material, just as I will include primary texts by representatives of the moral elites.

I cover a long period of time rather than describing in detail the moral elites of specific epochs. I will focus mainly on social policy ‘professionals’ rather than other potentially relevant groups such as authors of fiction (Kjældgaard, 2018). I limit my focus to particularly influential or illustrative individuals and groups.

I use civil society or third sector civil society as synonyms, but also private benevolence and philanthropy, depending on context. These concepts are inherently contested (Anheier & Knapp, 1990). What is important here is that they are designate forms of organizing social provision beyond social services delivered by state and municipalities (and market). The two ‘sectors’ involved in social services have never been neatly separated, but as ideals, images, or principles, the two have existed as separate entities—not least in the minds of the classifying elites.

## Moral Elites: De-paradoxifying State–Civil Society Relations Through Classifications

Paradoxes—“contradictory yet interrelated elements that exist simultaneously and persist over time” (Smith & Lewis, 2011, p. 382)—are challenging to logicians, but organizations routinely deal with paradoxes by *kicking the can down the road* (Luhmann, 2006) or by *carrying on as usual* despite contradicting orders (Luhmann, 2016). There are, however, also groups in society who do not simply live with paradoxes: planners, ideologues, and intellectuals—those that seek to create consistent and coherent visions for society—seek to eradicate them, to find expressions, formulas, and ideologies that can integrate, organize, and classify contradictions and in this way provide legitimacy for certain societal arrangements. I call these groups moral elites. Moral elites derive their *power from* control over certain resources, and in this way have the *power to* shape societal arrangements such as social policy.

### Moral Elites’ Power from: Education and Positions

Elite scholars typically define elites as groups that have “vastly disproportionate control over or access to a resource” (Khan, 2012, p. 362). Business elites have disproportionate control over the economy, political elites over parties, parliaments, and government, religious elites over the church etc. In contrast, moral elites have disproportionate access to a specific kind of resource, namely moral status or moral prestige. This resource is different from, e.g., economic resources, as it is mainly *derivative*. There is no one resource that lends moral authority to a group. Rather, moral authority is derivative of resources such as charisma, certain kinds of education (e.g., theology, medical schooling, law), or organizational top positions (church leaders, ethical commissions, professional associations, political parties).

Elite scholars have been reluctant to embrace the idea of a moral elite. Bottomore finds that only “on rare occasions have groups of intellectuals attracted any considerable public attention, or seemed to have a direct political influence” (Bottomore 1993, 57). However, the history of Danish social policy tells a different story: while the relative strength of groups in the ‘power elites’ (economic actors, political parties) was central for the overall direction of social policies, the principles behind changing social policy was not articulated by businessmen, but first by theologians, medical doctors, and lawyers, and later especially by economists with little access to direct power.

The Danish case also tells a story of waning and waxing moral elite status of certain groups. What lends moral authority to a group in one period may not suffice for another period. Mills has shown how business elites in 1950s USA cast themselves as a moral elite at the expense of other societal orders such as religion, school, and family (Mills, 1999). In this case, the ‘power elite’ was able to pay intellectuals to create an image of morality.

In Denmark, however, affluence does not really seem to have been a source of moral authority. Instead, education and affiliation with the state have been a constant for moral elite status. Changes in the moral elites have especially come about through the rising and falling ‘moral value’ of different types of university credentials: Denmark in the nineteenth century was dominated by a ‘traditional’ moral elite (Mannheim, 1980) embedded in pre-democratic, estate-based society, where groups such as Lutheran pastors, medical doctors, and lawyers had the moral authority to frame social policy. In Denmark, the rural elites became increasingly influential during the nineteenth century. Especially the large estate owners and the independent farmer class were influential in deciding the overall *direction* of social policy, since they would bear the brunt of the expenses through taxes (Baldwin, 1999). In the articulation and framing of policies in questions related to disease (e.g., tuberculosis), sexuality (e.g., prostitution), disability, elderly care, poverty, etc., the *moral authority* would lie with the educated classes that had been central in the administration of the absolutist state (Knudsen & Rothstein, 1994).

A central claim in this article is that while the paradox of conflicting principles in social policy became evident in the late nineteenth century, a moral elite with roots in the absolutist administration continued to exercise their influence over social policy across state and civil society. While I will describe shifts, dislocations, and circulations (Pareto, 1991) of elite groups—the fall of the clergy and the rise of the economists—much ideational change took place within the moral elite ‘estate’, the expert inheritors of the absolutist administration. As I will show and discuss in the conclusion, the moral elites were influenced by ideological currents in their time, but had clear agency in articulating these currents and framing social policy. It is thus an assumption of the article that moral elites enjoy ‘relative autonomy’ vis-à-vis the structural developments in society.

### Moral Elites’ Power to: Classification

Classifications in social policy are matters of general struggles over societal hierarchy (Bourdieu, 2018). Moral elites are crucial in defining who is deserving and what institutions should care for specific groups: Are charities or public welfare best suited to meet the needs of certain groups?

Through their work in politics, commissions, professional associations, journals, state administration, popular movements, philanthropic organizations, etc., changing moral elites have influenced criteria for deservingness and divisions of labor between public and private relief. Their task has not only been to change classifications, but also to defend (or in the words of Bourdieu: consecrate) existing social hierarchies, and in this way unfold, sidestep, overrule, or settle-for-now the paradoxes of conflicting principles in social policy.

First, moral elites are instrumental in classifying groups as deserving or undeserving. Perceptions of target groups matter immensely for the perceived legitimacy of social policies (Gilens, 1999). Factors such as the perception of a group’s control, attitude, reciprocity, identity, and need are crucial for public support for targeted social policies (Oorschot et al., 2017). Such criteria are, however, influenced by moral elites that have the authority to state that based on economic, theological, or medical grounds, a given group may in fact not be in control of its circumstances—which may change how this group is perceived. They may exercise this authority publicly (Schneider & Jacoby, 2005) or in policy framing processes (Campbell, 2002).

Secondly, and perhaps more importantly, the study goes beyond perceptions of target groups and shows the role of the moral elite in classifying the relation between sectors—and between sectors and deservingness perceptions. Scholars have pointed out the relation between charity and shame/stigma (Douglas, 1990; Ignatieff, 1984) and between shame/stigma and public relief (Gilens, 1999)—and shame and poverty as such (Walker, 2014). Moral elites have, however, been central in defining what kinds of provision should come with or without stigma.

To put in slightly more abstract terms: Individuals and groups as well as sectors may be classified by the moral elite as included or excluded, pure or impure, carrying stigma or not, being civil or noncivil, just or unjust (Alexander, 2006; Bourdieu, 1998).

**Fig. 1:**
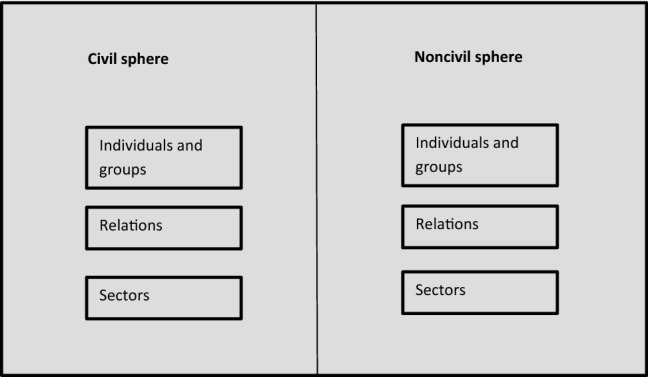
Alexander’s model of civil and noncivil spheres

Figure 1 builds on Alexander’s idea that there is in society such things as civil and noncivil spheres that expand and contract historically so that at any given time, certain groups are considered deserving, some relations good, and some institutions beneficial—while others are the opposite. Who is in an and who is out depends on historical contingencies. I claim that moral elites have a stake in deciding who and what are in and out of the sphere. I will refer to the figure throughout the article.

## Three Historical Classifications

I will now show how moral elites have struggled over the classification of social policy in Denmark in order to de-paradoxify the contradictory principles of philanthropy and rights that co-existed from c. 1849 until today. The analysis falls in three parts: Help to self-help (1849–1891), Rights classification (1891–1976), and Workfare classification (1976-present).

### Traditional Moral Elites and the Help to Self-help Classification (1849–1891)

The latter half of the nineteenth century saw the emergence of a new moral elite, or perhaps better: of new roles to existing moral elites. Education within religion, law, and medicine had long secured influence on the state’s social policy under absolutism: the commission behind the “Enlightenment” poor law reforms of 1799 and 1803 were recruited from church, local, and central administrations (Johansen & Kolstrup, 2010, p. 181). However, with the economically liberal currents that swept across the country with the 1849 constitution and the 1857 freedom of trade act and break-down of guilds, poverty took on new proportions and forms, and the traditional elite would take up tasks in civil society.

The civil society involvement of the traditional moral elites grew from their experiences with prison inmates (MD Christian Geill (1860–1938)), and from their responsibilities as leaders and caretakers of parishes, including figures such as theologians and pastors J.C. Holck (1824–1899), Vilhelm Munck (1833–1913), N.C. Dalhoff (1843–1927), Harald Stein (1840–1900), and later Alfred Th. Jørgensen (1874–1953) who were connected to philanthropic institutions such as *Diakonissestiftelsen*, The Copenhagen Home Mission, and the Stefanus Association.

The new forms of poverty and new organizations brought the social policy paradox to the fore: Who were deserving, and should private or public institutions care for them? Moral elites would coalesce around the principle of help to self-help. Each sector would play its role in a shared consensus across the public/private divide on how to solve social problems (Bundesen et al., 2001, p. 402f). Help to self-help entailed deterring the poor from public relief and encouraging them to become self-reliant.

The public system should care only for the undeserving, the lazy and unwilling. In principle, the undeserving needed to feel the natural consequence of laziness, namely hunger, and if the public system were to support them, they should be put to hard work and lose their political and civil rights. Meanwhile, private philanthropy was expected to care for the mere unfortunate, the deserving whose morality should not be undermined by being cared for with the undeserving (Dalhoff, 1900, p. 134f).

Traditional moral elites agreed on criteria for deservingness (bad fortune, but individual willingness to become self-reliant), but the question remained how to apply the criteria. Moral elites were increasingly critical of the state’s ability to discriminate between different groups of poor people and instead looked to civil society. The philanthropic Christianshavn Relief Society (est. 1866) developed standardized forms to judge whether a person was deserving or not: first, a questionnaire was filled out by the applicant. The applicant was required to answer five questions about their family status, other types of support they might be receiving, and the type of employment they were hoping to gain. Then, one of the organization’s volunteer investigators would check if the answers were truthful and answer another 12 questions related to place of residence, ability and desire to work, types of income, health, etc. (Christianshavns Understøttelsesforening, 1866). Volunteers conducted home inspections to establish whether or not the poor had a legitimate claim or not (Munck, 1869, pp. 68f, 78f). The principles of Christianshavn Relief Society were copied in several other urban areas in Denmark, and the Copenhagen Relief Society was founded in 1874 on similar principles (Nørgård, 2015, p. 227). Only civil society was trusted to carry out classifications.

However, from the traditional elite’s own ranks, groups would emerge that did not resolve the social paradox simply by classifying according to the existing consensus, but further wished to push groups between categories. They, too, saw civil society as superior to state benevolence. In fact, some envisioned a radical civil society strategy, where an activist, decentral church driven by zealots would emerge from within the national church, take over social and moral responsibilities, and eventually render the state church and state-organized social services obsolete. The group was influenced by new radical revivalist ideals stemming from the Reformed (Calvinist) tradition as well as medical-scientific ideas about the impact of heredity in certain of the groups otherwise considered undeserving. In Copenhagen, national economists (H. Westergaard, 1853–1936), Egyptologists (H.O. Lange, 1863–1943), as well as medical doctors (P.D. Koch, 1856–1941), and pastors (H.P. Mollerup, 1866–1929, H. Ussing, 1855–1943, J.F. Hansen, 1856–1905) constituted an educated elite that was less rooted in existing institutions. They developed ideals for the self-organization of the congregation, founded abstinence associations, promoted labor protection such as resting on Sundays (*Foreningen til Fremme af Søndagens rette Brug*), and organized the Church Army.

This sub-elite combined their civil society strategy with scientific ideas in order to reclassify groups that had been deemed morally flawed and left to the public system: These groups did not lack moral character but were victims of inherited degenerative traits triggered by a normless urban environment and a de-Christianized society. Alcoholics constitute the prime example of a group that this moral elite targeted through the abstinence associations and treatment facilities—based on religious and scientific ideals (Sevelsted, 2019).

**Fig. 2:**
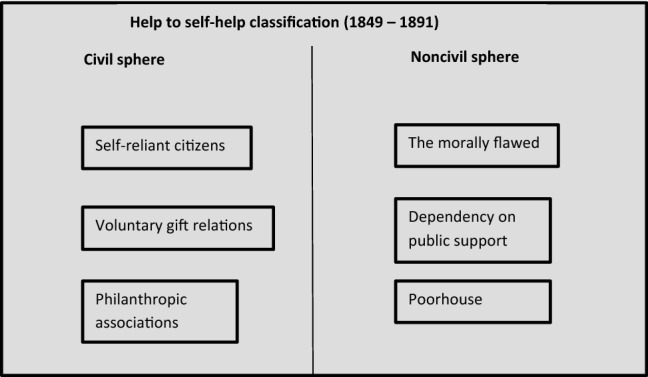
Help to self-help classification

The help to self-help classification remained the central way of handling the social policy paradox: Benevolence was divided into a public and a private branch which each had a role in supporting, encouraging, surveilling, disciplining, educating, and punishing the poor. The sectorial division of labor seemed clear, and only the discussion over groups’ deservingness remained (Fig. 2).

However, the ‘scientification’ and specialization of the social question would in turn—paradoxically—pave the way for the state-enthusiastic moral elite of the mid-twentieth century.

### Specialists and Rights Classification (1891–1976)

The twentieth century was marked by a change in the composition of moral elites and their allegiances, and thus also in their classifications. The professionalization and specialization of social policy and social work started by revivalists within the traditional moral elites was continued as the state was revalued as a social provider.

A new generation within the moral elite of national economists, social policy experts, and eventually social workers and similar professions would discuss social policy in specialized associations, journals, research centers, and committees (i.e., Association for Social Policy (*Socialpolitisk Forening,* 1898), ‘The Social Journal’ (*Socialt Tidsskrift,* 1925), The Social Research Institute (*Socialforskningsinstituttet,* 1964), and The Social Reform Committee (1964)—and increasingly, inter-national organizations such as the *Nordic Council* would become fora for moral elites to organize and communicate. These were the ‘social engineers’ that by the 1930s would dominate social policy (Kolstrup, 2011)—engineers that would increasingly be integrated with the politically dominating Social Democracy. As the church was deprived of its former administrative role in poor relief, the clergy would lose influence within the elite (Sevelsted, 2020).

Just as revivalists of the nineteenth century had argued that few should be considered undeserving, so did this new elite—only now they argued for a role-reversal between private and public relief. The old age pension law of 1891, introducing universal old age pension (albeit with a ‘moral clause’), marked the first step toward the ‘rights classification’ that would become the consensus with the social reform of 1933 and reach its zenith with the Social Assistance Act of 1976 (*bistandsloven*). Now, private charity was considered uncivil. A rights-based social system would be developed that sought to cover any and every need of citizens, while leaving it to the discretion of the social worker to decide what benefits citizens should receive.

Lawyer and future Social Democratic minister of the interior and social affairs, K.K. Steincke, famously entitled a 1912 book “Alms or Rights” (Steincke, 1912). Steincke paved the way for a structured social policy in the 1930s. In his view, it was not the public system but charity that contributed to demoralizing the population (ibid., p. 18). However, certain areas of social policy were best taken care of by philanthropy and its ‘warm interest in the individual’: care for children, vagrants, the mentally ill, prostitutes, and alcoholics. Philanthropy, importantly, should be supported and supervised by the public system (Steincke, 1920, pp. 391–402). Now, charity was considered demoralizing, and only the public system would secure the dignity of the working class through the politics of the ‘social minimum’. This reassessment would be further developed by the next generation of the welfare state’s moral elites.

Julius Bomholt (theologian and Social Democratic minister of education, social affairs, and culture, 1896–1969), Jørgen S. Dich (Professor of economy, 1901–1975), Henning Friis (economist, 1911–1999), and Bent Rold Andersen (Professor of economy, Social Democratic minister of social affairs, 1929–2015) were after WW2 deeply involved in heralding a new era in which social experts closely connected to the Social Democratic Party would work to reclassify institutions and social groups. Bomholt spearheaded the Public Care Act of 1961 (*Forsorgsloven*). He advocated “fellow citizen help” (*Medborgerhjælp*) to replace existing forms of social relief. This meant that the last references to deservingness in social law were stricken, and public support from now on did not result in loss of political or civil rights (Kolstrup, 2012, pp. 174–183). State bureaucracy continued the professionalization and differentiation of special care spearheaded by nineteenth-century religious philanthropists, increasingly moving groups away from general support and into specialized treatment (ibid., 183). The Social Reform Committee (1964) became a hub for this new moral elite of Social Democratic social experts developing a comprehensive approach to helping the individual adapt to a changing social environment—a development that culminated in the Social Assistance Act of 1976 (*bistandsloven*). The individual was not to blame for their social situation; instead, social problems should be considered the result of societal factors and a still imperfect public social assistance system that needed to be developed to assist individuals who had been affected by social misfortune (ibid., 189ff; Åkerstrøm Andersen, 2008; Knudsen, 1985).

Philanthropy was now considered entirely inadequate and basically a means to achieve absolution for a guilty conscience. Philanthropic endeavors were forced to adhere to specific standards and norms if they were to act as service providers for the public system. Others were taken over by the public, crowded out, or forced to change their bylaws to become ‘self-governing institutions’, essentially foundations with a broad representation on their boards (Kolstrup, 2012, p. 205). The clergy of the traditional moral elite would survive within philanthropy—now saluting the welfare state as a “God-given arrangement” (*en Guds ordning*) (Malmgart, 2010, p. 57). Social scientist Michael Ignatieff’s sentiment captures the general ideology of the period: “bureaucratized transfer of income among strangers has freed each of us from the enslavement of gift relations” (Ignatieff, 1984, p. 17).

**Fig. 3:**
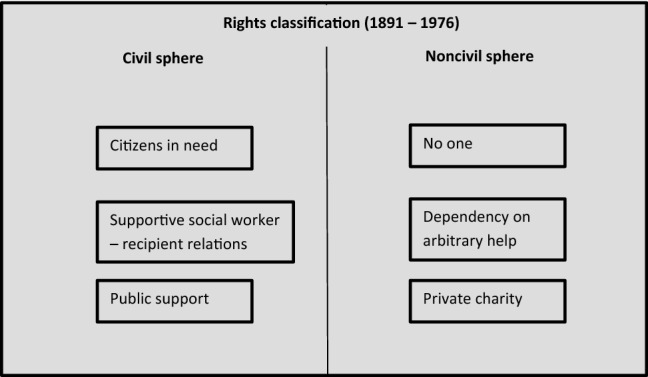
Rights classification

The welfare state’s moral elites of social economists and social policy experts were closely connected to the Social Democratic party and became influential through designated associations, journals, research institutions, and committees. They were economist in a sense that is almost forgotten: social policy experts who develop plans for how different types of expertise could help marginalized individuals become self-supporting. The paradox of conflicting logics of social policy was pushed into the future: better planning would eventually eliminate the need for philanthropy, and philanthropy was thus demoted to the subordinate position of ‘service provider’ or ‘pioneer’ (Fig. 3).

As in the first period, in an almost Hegelian fashion, the long build-up to the welfare state carried with it its own internal contradictions and struggles in embryonic form. From the anti-statist left as well as the anti-statist right, critics of the state-professional solution to the paradox eventually emerged (Petersen et al., 2012, p. 147ff).

### Economists and Workfare Classification (1976–present)

During the third period, the composition of the moral elite within social policy would change so that social policy experts would retreat in favor of a new type of economist focused on labor market participation. This new economist elite emerged largely as the result of an ideational change within the existing elite that was growing increasingly skeptical of the state—a skepticism voiced in the committees, councils, journals, and associations that had been develop under the rights classification.

Just as the welfare state reached its zenith, its value would drop rapidly in the eyes of the moral elites. Jørgen Dich—mentioned above as one of the architects of the comprehensive state approach—would in 1973 publish a book called *The Ruling Class*, arguing that civil servants had de facto turned into a ruling class, exploiting the public sectors for their own purposes (Dich, 1973). The Social Research Institute was now questioning the comprehensive social system (Thorlund Jepsen et al., 1974). At the institute, a new generation of scientists revalued civil society based on its distinct motives, its flexibility and its advocacy vis-a-vis the public system (Boolsen, 1988) or—like the revivalists of old—envisioned a counter-public through which the everyday lifeworld would be able to self-organize in opposition to the state apparatus (Habermann, 1990). This resonated with the way the traditional moral elite in Christian philanthropy continued to view philanthropy as carried by personal commitment and neighborly love (Kristophersen, 2020). A number of reports would be produced during the 1980s to reappraise the strengths of the third sector (Boolsen, 1988; Habermann & Parsby, 1987; Jensen et al., 1987; Jeppesen & Høeg, 1987). Eventually, the laissez-fare liberal think tank CEPOS (Center for Political Studies) (est. 2004) would herald civil society as a benevolent provider of relief (Gade Jensen, 2011). Increasingly, civil society was viewed as a solution to what OECD in 1980 had dubbed “the crisis of the welfare state” (OECD 1981).

The labor market was itself reclassified to make room for an ‘alternative’ or ‘third’ labor market where the ‘least employable’ would be able to find work. This idea has also been launched under the idea of a ‘fourth’ sector of for-profit socio-economic businesses (Levitt, 2012). Increasingly, commission reports and civil society strategies argue for ‘partnerships’ between the private and public sector in order to get the best from both worlds (Jessen, 2019).

The reclassification of sectors went hand in hand with a reclassification of target groups who again were considered according to their moral-economic habitus rather than as victims of economic fluctuations. Social experts developed this approach in close collaboration with new Social Democratic politicians inspired by Tony Blair’s ‘third way’ (Petersen et al., 2014). Especially two commissions helped legitimize the new approach: the Social Commission (est. 1991) and the Zeuthen Commission (est. 1991), dominated by economists (Olesen, 2004). The commissions focused mainly on unemployment, but their work entailed a renewed focus on the labor market as the solution to social problems, and thus a closer integration of labor market and social policy. Work would now seize to be only a goal; it was also a means in social policy. Passivity became the enemy and activity the goal—activity that was eventually meant to lead to employment. In time, a kind of second-order classification consensus emerged according to which specialized classifications by professionals such as medical doctors or psychologists were subsumed under economistic classifications of individuals’ ability to work measured in percentages (*arbejdsprøvning*) (Hansen, 2017, pp. 107–134).

**Fig. 4:**
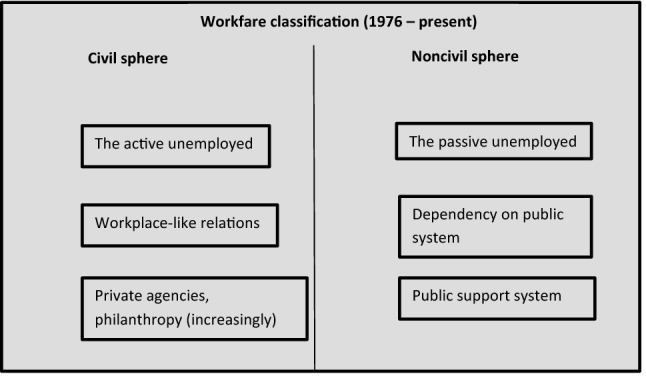
Workfare classification

The role reversal of sectors is complete. In the first period, private philanthropy discriminated between the unfortunate and the lazy. Now, this is the job of the public sector, while charity takes the rest—as did the nieneteenth-century state. As we see, the relationship between perceptions of deservingness and perceptions of sector responsibility is not stable, but fluctuates with the classification of groups as well as sectors (Fig. 4).

## Conclusion: Moral Elites and the Paradoxical Classification of Social Policy

The analysis of the three eras of classification shows that perceptions of social policy target groups and of sectors in social provision cannot be separated. Public and private support have coexisted since before the nineteenth century as a paradox of “contradictory yet interrelated elements” in social policy. Changing moral elites have been central in de-paradoxifying this relation through hierarchical classifications. To do this, such elites have drawn their *moral power from* different resources and used their *moral power to* classify in different ways.

The late nineteenth-century moral elite drew their authority from their status within the old estate society. Absolutism relied on the clergy, medical doctors, and lawyers to develop social policies. From within these ranks rose the policy professional—economists, social scientists, social workers—that during most of the twentieth century would draw on specialized knowledge for their moral elite status. They would establish specialized research institutions, specialist journals, and associations. The social policy experts continue to dominate social policy, but economists have ascended to the top of the moral elite hierarchy as other types of knowledge are increasingly measured against their economic utility, shown by the fact the department of finance has become *the* dominant department in the central administration.

In order to understand how the social policy paradox is settled in different historical contexts, it is necessary to distinguish different kinds of classificatory strategies. The most basic is the strategy of *classifying individuals*. The first home inspectors of the nineteenth century were equipped with forms to help them determine if an individual was deserving or not, and every social worker since has been tasked with this job. A more demanding classificatory effort is involved in the *reclassification of groups*. Nineteenth century religious zealots and medical doctors worked to push groups such as prostitutes and alcoholics from the undeserving to the deserving category. Today, ideological battles are fought over the status of the unemployed. Related to this is the classificatory strategy of *reclassifying sectors*. In the nineteenth century, support from the public sector was intentionally stigmatizing while in the twentieth century, philanthropy would be framed as stigmatizing. These different classifications depend upon *classifications of relations*, i.e., what relations are viewed as civil or noncivil, demeaning or proper? Is it more demeaning to rely upon ‘passive’ state income transfer, on ‘paternalistic’ charitable foundations, or to be enrolled in public activation programs?

Presently, economists have gained a ‘second-order’ moral authority of classification (a ‘classification of classifications’). This means that economists increasingly have the last word vis-à-vis social workers, social policy experts, or medical doctors’ classifications. Increasingly, classifications of groups’ deservingness and sectors’ civility are measured against their economic utility: No sector is inherently civil or noncivil if it can contribute to the activation of individuals.

The case of Denmark shows both the connectedness and divergence of moral elites and their classificatory efforts. As a small nation state, Denmark has to some extent functioned as a weathervane for international ideational trends in the moral elites. Nineteenth-century Christian philanthropists were inspired by the German *Innere Mission* as well as UK Christian socialism (Sevelsted, 2018, 2021), and the high moral assessment of the state as a provider of services after the second world war, and its devaluation from the 1980s, are similarly inter-national trends underpinned by moral elites’ classification efforts (de Swaan, 1988; Offer, 2006). However, a look at the moral elite also reveals how national trajectories differ. In nineteenth-century UK, discussions over the role of different sectors in social policy took place among university trained philosophers (Offer, 2003). In Denmark, this was only the case to a lesser extend (see, however, Høffding (1887)) where the traditional absolutist moral elite of pastors, medical doctors, lawyers, and eventually economists were key in influencing principles of classification. Perhaps the developmental path of Danish social policy can in part be explained by the fact that here the moral elite was more the elite of an administrative craft inherited from absolutism than of a discipline within the humanities. Similarly, the present institutionalization of neo-classical economic ideas has not resulted in sudden social policy budget cuts, but rather in using the state bureaucracy to incentivize individuals according to these ideas (cf. Stahl, 2019).

Focusing on the role of moral elites in the development of changing classificatory regimes opens new avenues in studies of perception of deservingness in social policy. First, it would put focus on who shape policies as well as public opinion. Moral elites are often close to the power elite and help frame elite discussions over social policy. Moral elites also shape public perception of target groups - perceptions of groups control, attitude, reciprocity, identity, and need (CARIN) (Oorschot et al., 2017). Moral elites such as medical doctors and economists often work as opinion leaders (Schneider & Jacoby, 2005) in shaping public perception: Whether the chronic alcoholic is viewed as in control or not in control of her/his situation may depend on whether alcoholism is considered a personal flaw or a disease, and economists may shape perceptions of the unemployed, depending on their Keynesian or neoliberal leanings. Such an approach would add to de Swaan’s focus on elites’ material interests and interdependence (de Swaan, 1988).

Second, while there is scholarly focus on the classification of individuals and target groups in social policy, less attention has been paid to the relationship between the classification of target groups and the classification of sectors. Target groups may be considered deserving poor while at the same time, they are thought to be helped through third sector associations—exactly because the public sector is perceived to be a last resort that should only care for the undeserving. While scholars such as Douglas (1990), Ignatieff (1984), Gilens (1999), and Mau (2014) have directly or indirectly touched upon the stigma of either private or public provision, the historically varying relation between the two remains in the dark. The study of changing sector classifications opens for increased understanding of the relations between perceptions of deservingness and perceptions of sectors.

There are now some indications that the dominance of the active labor market classification and the reign of the economists may be challenged in many Western countries: Increasing nationalism, the crises of climate, COVID-19, and in some respects also the crisis of a workfare regime, where some unemployed mobilize against demands for activation, seem to call for more state involvement. Economic historians have noted that in Denmark, perhaps following an international ‘populist’ trend, political scientists have replaced economists atop the moral elite hierarchy, indicating a shift in moral categorizations (Hansen et al., 2019). In this new situation, what moral elites will now de-paradoxify social policies? What resources will they rely on and how will they classify?
